# Distinguishing the Biomass Allocation Variance Resulting from Ontogenetic Drift or Acclimation to Soil Texture

**DOI:** 10.1371/journal.pone.0041502

**Published:** 2012-07-24

**Authors:** Jiangbo Xie, Lisong Tang, Zhongyuan Wang, Guiqing Xu, Yan Li

**Affiliations:** 1 State Key Lab of Desert and Oasis Ecology, Xinjiang Institute of Ecology and Geography, Chinese Academy of Sciences, Urumqi, Xinjiang, People’s Republic of China; 2 Graduate School, Chinese Academy of Sciences, Beijing, People’s Republic of China; Pennsylvania State University, United States of America

## Abstract

In resource-poor environments, adjustment in plant biomass allocation implies a complex interplay between environmental signals and plant development rather than a delay in plant development alone. To understand how environmental factors influence biomass allocation or the developing phenotype, it is necessary to distinguish the biomass allocations resulting from environmental gradients or ontogenetic drift. Here, we compared the development trajectories of cotton plants (*Gossypium herbaceum* L.), which were grown in two contrasting soil textures during a 60-d period. Those results distinguished the biomass allocation pattern resulting from ontogenetic drift and the response to soil texture. The soil texture significantly changed the biomass allocation to leaves and roots, but not to stems. Soil texture also significantly changed the development trajectories of leaf and root traits, but did not change the scaling relationship between basal stem diameter and plant height. Results of nested ANOVAs of consecutive plant-size categories in both soil textures showed that soil gradients explained an average of 63.64–70.49% of the variation of biomass allocation to leaves and roots. Ontogenetic drift explained 77.47% of the variation in biomass allocation to stems. The results suggested that the environmental factors governed the biomass allocation to roots and leaves, and ontogenetic drift governed the biomass allocation to stems. The results demonstrated that biomass allocation to metabolically active organs (e.g., roots and leaves) was mainly governed by environmental factors, and that biomass allocation to metabolically non-active organs (e.g., stems) was mainly governed by ontogenetic drift. We concluded that differentiating the causes of development trajectories of plant traits was important to the understanding of plant response to environmental gradients.

## Introduction

Biomass allocation is a central issue in plant life history [1], and provides the basis for understanding the response or adaptive strategies of plants [2]. Different biomass allocation patterns reflect how plants respond to different selection pressures [2]. Plant biomass allocation patterns depend primarily on genetic differences between species [2]–[4]. Adjustments in biomass allocation can also be plastic as a response to varying resource availabilities [5]–[8], or as a result of ontogenetic drift [1], [9], [10] or both [9], [11], [12]. However, it is difficult to distinguish the variation in biomass allocation due to environmental gradients versus ontogenetic drift [9], [13], [14].

Evans [15] defined ontogenetic drift as changes of a biological trait in such a predictable way that it can be presented as a function of plant growth or development [9]. Biomass allocations to leaves, stems and roots have been intensively investigated from this perspective. For annual plants, it was found that the proportion of allocation to roots declines during their development [9], [11], [16], but for perennials, relative biomass allocation to belowground (i.e. roots) was proposed to increase as they grow up [17], [18]. Therefore, some studies have concluded that biomass allocation pattern to different organs is size-dependent, i.e., results from ontogenetic drift [9], [18], [19].

In contrast, the functional equilibrium hypothesis regards plant biomass allocation as size-independent [2], [9], [14], [20], [21]. It suggests that plant will develop larger root system if soil resources are limiting, and will allocate proportionally more to stems and leaves if an aboveground resource such as light is limiting [8], [22]–[29]. The goal of plastic response to environment is for plants to maximize growth and survival under resources limitation [3], [30]–[33]. Therefore, if soil water is limiting, plants must develop more absorbing roots and less leaf area to maintain balance between water absorption and consumption [34]–[36]. But support organs such as stem plays no roles in resource acquisition in most terrestrial plants and thus should not respond to resource limitation. Thus studying the absorbing organs and support organs separately is necessary if a full understanding is desired [23], [24].

However, changes in biomass allocation attributed to environmental variations may also result from ontogenetic drift [2], [9], [14], [18], [24], [37], [38]. Some studies even suggested that observed plasticity in biomass allocation may be due only to ontogenetic drift [9], [16]. Is the variation in biomass allocation a result of ontogenetic drift or acclimation to environment [9]? To date, conclusions from the literature are ambiguous. In a review, Niklas [39] suggested that these two explanations were not mutually exclusive: development of different organs and their traits are also the objects of environmental selection. However, if plants develop along different trajectories in contrasting environments, understanding how organs develop will require distinguishing the result of ontogenetic drift or response to the environment [9], [13], [14], [24], [40], [41]. Nevertheless, few studies have distinguished the roles of differences in size (ontogenetic drift) and differences in developmental trajectory (response to environment) as the basis for allocation responses to the environment.

In a previous study, we found that the soil texture significantly affected the biomass allocation of cotton plants under well-watered conditions [34]. Here, using a similar controlled experiment with contrasting soil textures, we tried to determine whether the biomass allocations to different organs were mainly governed by ontogenetic drift, soil texture or both.

## Materials and Methods

### Creation of Grades in Soil Texture

**Figure 1:**
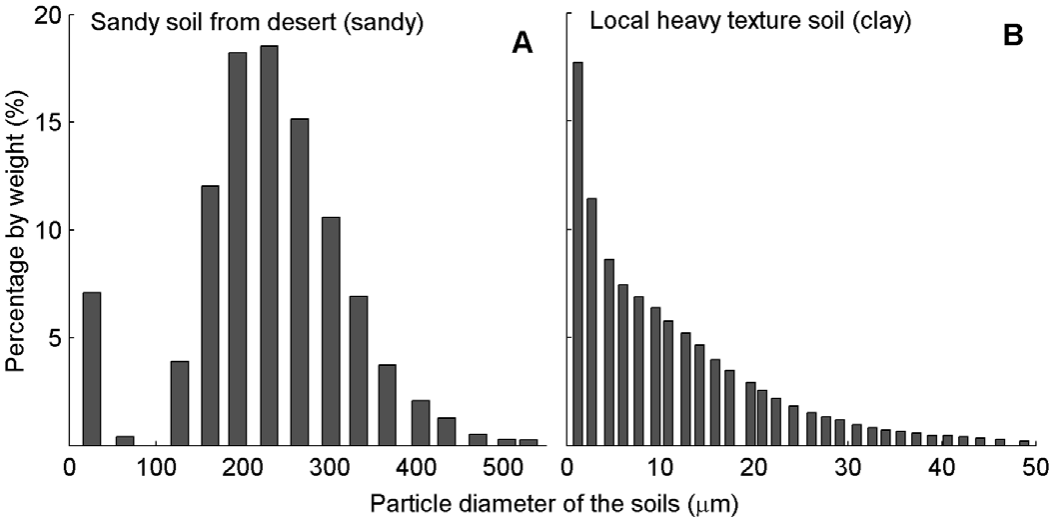
Particle size distribution of the two soil textures. (A) Sandy soil. (B) Clay soil. Data were obtained from particle size measurement by a laser diffraction system (Sympatec GmbH, System-Partikel-Technik, Clausthal- Zellerfeld, Germany).

The experiment was carried out during the cotton-growing season of 2010 at the Fukang Station of Desert Ecology, Chinese Academy of Sciences – located on the southern periphery of the Gubantonggut Desert, in the hinterland of the Eurasian continent (87°56′ E, 44°17′ N, 475 m a.s.l.). Two grades of soil texture were created by taking local fine-textured soil and nearby desert sand. The particle size distributions of the desert sand (hereafter referred to as sandy) and the local fine-textured soil (hereafter referred to as clay) were determined by a laser diffraction system (Sympatec GmbH, System-Partikel-Technik, Clausthal- Zellerfeld, Germany) (Fig. 1). The particle diameters of the sandy soil were <500 µm and that of the clay were <50 µm (Fig. 1). The soil textures gradients are based on the root contact, which create a partial physical discontinuity at the soil–root interface for movement of water and nutrients from soil to roots. Therefore, sandy soil treatment used in this study would have lower availability of resources than clay soil treatment [34].

### Plant Materials and Measurement of Plant Traits

**Figure 2:**
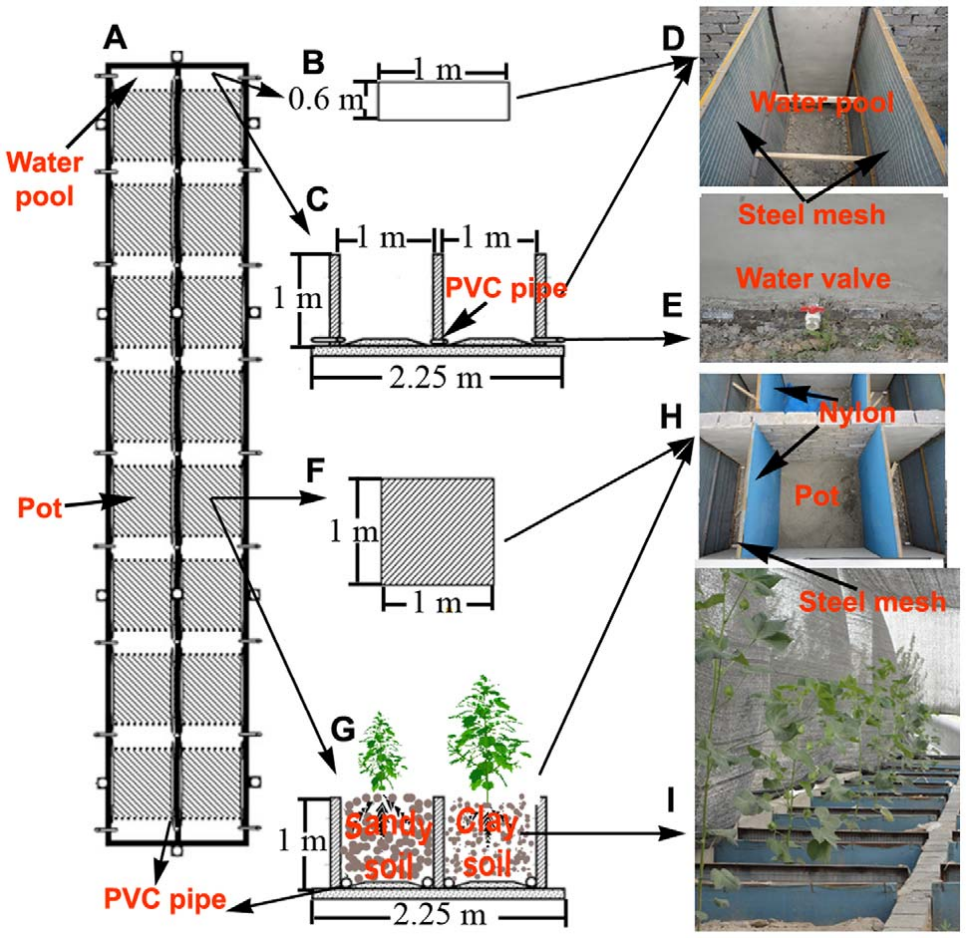
Schematic diagram of the experiment. (A) Plan form of experimental device; (B) plan form of water pool; (C) cross section of water pool; (D) photograph of water pool; (E) water valve; (F) plan form of pot; (G) cross section of pot; (H) photograph of pot; and (I) photograph of experimental plants. The experimental devices (A) were constructed by cement. Each device was divided into 2×8 cells. Each cell contained a pot and a water pool. Each pot (1 m ×1 m ×1 m) was located between two water pools (0.6 m ×1 m ×1 m). The steel mesh (covered with nylon) was used to separate the pot from the water pools, and all water pools in the device were connected by PVC pipes (the length of PVC was 1.2 m; A, G). The left pools were also connected with right pools by PVC pipes (C). Thus the water could run-through the whole device. The left pots were filled with sandy soils, and the right pots with clay soils (G, I).

A total of 150 pots (1 m × 1 m × 1 m) were filled with either sandy or clay soils, with 75 pots per soil texture (Fig. 2). Cotton (*Gossypium herbaceum* L., Variety: Huai-Yuan 170) seeds were sown on 15 May 2010, and only one plant was left to grow in each pot after emergence. All pots for each soil texture were placed in an open field. Prior to sowing and after filling the pots with soil, all pots were watered and drained continuously for 10 d in order to wash out salt or nutrients. All potted plants were kept well-watered and treated with pesticide to avoid physiological stress during the whole growing period. Soils in arid region are generally nutrient-poor. To avoid nutrient deficiency during plant growth, all pots were irrigated frequently (water filled the experiment devices once a week for both soil textures, and water amount was measured by water meter; Fig. 2) with a modified Hoagland nutrient solution (0.4 NH_4_H_2_PO_4_; 2.4 KNO_3_; 1.6 Ca(NO_3_)_2_; 0.8 MgSO_4_; 0.1 Fe-EDTA; 0.023 B(OH)_3_ [boric acid]; 0.0045 MnCl_2_; 0.0003 CuCl_2_; 0.0015 ZnCl_2_; 0.0001 MoO_3_; all concentrations in units of millimoles per liter of water). Then drained after 5 h. Prior to fertilizing, all pots were watered and drained continuously for 5 h in order to wash out fertilizer residue of the previous application.

Plants were harvested and measured four times at 30, 40, 50 and 60 d after germination (before reproduction stage), with 15 randomly selected replicates for each soil at each time.

The measurements consisted of plant height (*H*) and basal stem diameter (*D*) [42]. Furthermore, leaves and roots (intact root systems of cotton were excavated to determine their length) were scanned into images [36], and leaf area (including green petioles) and root length were computed by CI-400 CIAS (Computer Imaging Analysis Software; CID Co., Logan, UT, USA). Then plants were divided into roots, stems (including non-green petioles) and leaves (including green petioles), which were dried to a constant weight, and root mass (*M_R_*), stem mass (*M_S_*) and leaf mass (*M_L_*) were measured. Thus, total plant size (*M*) was calculated as *M* = *M_R_*+*M_S_*+*M_L_*. Root mass ratio (*RMR*), stem mass ratio (*SMR*) and leaf mass ratio (*LMR*) were calculated as the ratio of roots, stems and leaves, respectively, to *M*. Specific leaf area (*SLA*, leaf area per leaf mass) and specific root length (*SRL*, root length: root mass) were also calculated [23].

### Data Analysis

The data were categorized according to plant size and age after germination to evaluate the effects of plant size and age on variables of interest. Five classes of plant size categories were defined in body size: i.e. A <1 g, 1 g ≤ B <5 g, 5 g ≤ C <10 g, 10 g ≤ D <20 g and E ≥20 g. Age was defined simply as ‘days after germination’.

**Figure 3:**
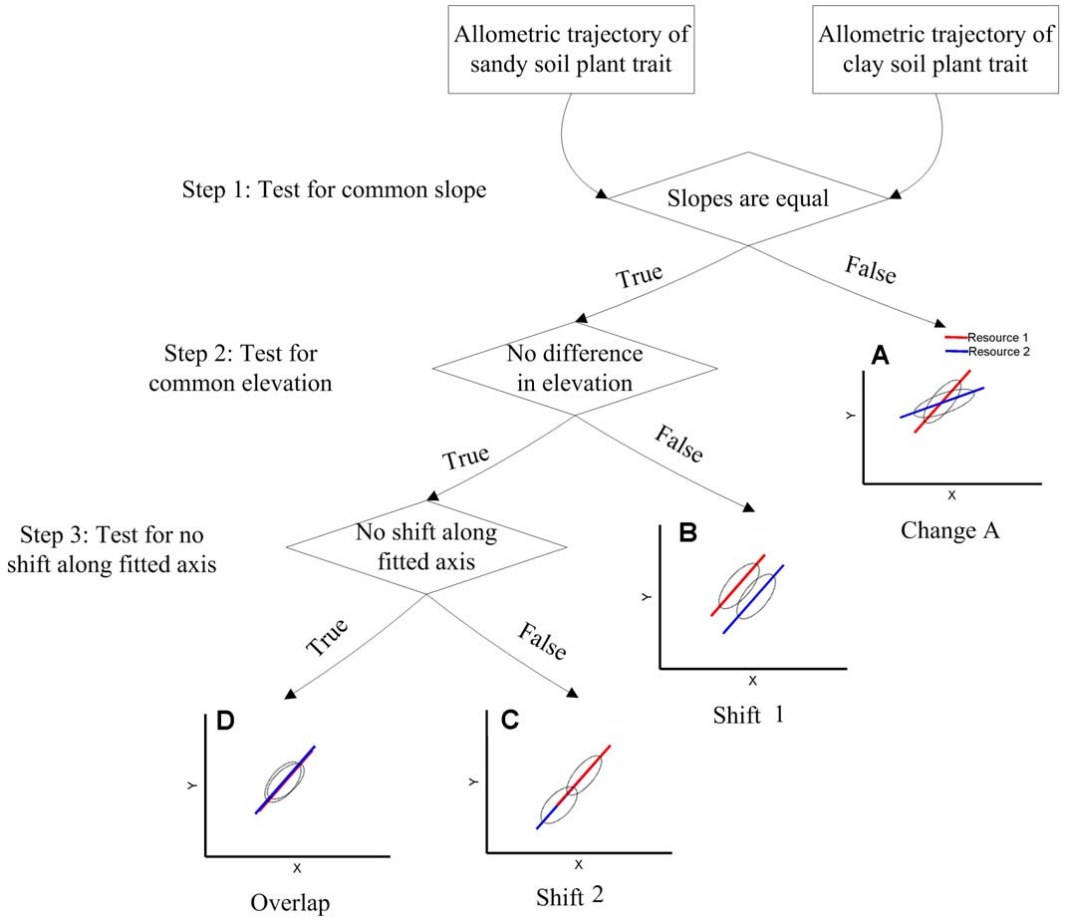
Three steps and four types in making inferences about two lines. Four types: (A) Change is defined as the slopes are not equal in the two soil textures; (B) Shift 1 is defined as the slopes are equal but the intercepts are differences in the two soil textures; (C) Shift 2 is defined as the slopes are equal, and the intercepts are no differences in the two soil textures, but are shifted in location along common slope SMA lines; (D) Overlap is defined as no difference in slopes, no difference in intercept and no shift location along common slope SMA lines.

Standardized (or reduced) major axis (SMA or RMA) regression analysis was used to test the *log10* ^Y^ *− log10* ^X^ scaling relationship and the SMA slope (parameter *b*) or intercept (parameter *a*) heterogeneity between soil textures using the Standardized Major Axis Estimation and Testing Routines (SMATR) package of R (R version 2.13.1, R Foundation for Statistical Computing) [43]–[45]. SMA regression was used in exploring the relationship between two plant traits (Y and X) changes along the environment gradients. There are three steps in testing the *log10* ^Y^ *− log10* ^X^ SMA lines from plants in two soils (Fig. 3; data used in Fig. 3 for illustrative purposes only). There are four possible results in the testing: (i) the two SMA lines may have different slopes (Fig. 3A, this suggests that different soil textures significantly changed the developmental trajectories of plant trait. Hereafter referred to as type Change). Otherwise, if the two SMA lines share a common slope, the two SMA lines may (ii) be different in intercept (Fig. 3B, this suggests that plant in different soil textures have different trait, for a given plant size. Hereafter referred to as type Shift 1); (iii) share a common intercept but be shifted in location along a common slope SMA line (Fig. 3C. Hereafter referred to as type Shift 2); (iv) No difference in intercept and no shift location along common slope (Fig. 3D. Hereafter referred to as type Overlap).

**Figure 4:**
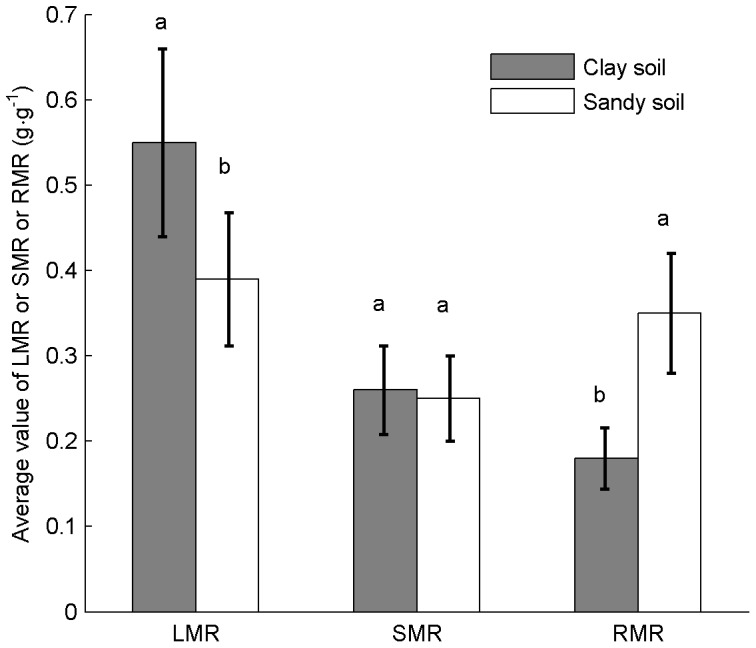
Average values of *LMR*, *SMR* and *RMR* in the two soils over ontogeny. Bars represent average values ± CV, n = 60. Different letters indicate differences (*p*≤0.05) of biomass allocation to leaves, stems and roots between the two soils.

Different slopes and/or different intercepts show that the relationship between the two variables or biomass allocation is affected by soil textures (type Change and Shift 1). Equal slopes and intercepts among treatments show that the relationship between the two variables or biomass allocation remains the same in different soil textures (type Shift 2 and Overlap) – in other words, biomass allocation is only a function of plant size and changes in allocation were explained by ontogenetic drift [24]. In addition, the type Shift 2 (shifts in slope) would also suggest that the quantitative change of plant phenotypes in a resource-poor environment is simply due to developmental delay.

We also wanted to distinguish between the influence of soil texture and ontogenetic drift on biomass allocation. For this purpose, nested ANOVAs were performed to partition the variance components of *LMR*, *SMR* and *RMR* across soil gradients and plant size categories (Nested Procedure, SAS version 8.0; SAS Institute Inc., Cary, NC, USA) [46].

Nested or hierarchical designs are commonly used in environmental effects monitoring studies. In this study, one level was groups of different soil textures; while another level was subgroups, i.e. the different plant size categories; and finally within subgroups, i.e. the replications in each plant size categories. We converted the measurement variables (*LMR*, *SMR* and *RMR*) to ranks (rank transformation, replaced each observation with its rank over the entire data set). So the variables were partitioned into subsets, and each subset was ranked within itself independently of the other subsets [47]. If the biomass allocation pattern variance only resulted from a delay in growth alone, the plants of the same size (i.e. the same developmental stage) would have a similar biomass allocation pattern [24]. Thus we used nested ANOVAs on the ranks step-by-step: first between A and B plant size categories, second on B and C plant size categories, and then on C and D and so on. The nested design allowed us to test two things: (1) difference among groups (soil textures), and (2) the variability of the subgroups (plant size categories) within groups (soil texture). If we failed to find significant variability among the subgroups within groups, then a significant difference between groups would suggest an environmental impact – in other words, the variability was due to differences in soil texture and not to plant size categories. In such situations, however, it was highly likely that there would be variability among the subgroups. Even if this was significant, we could still test whether the difference between the soil textures was significantly larger than among plant size categories.

## Results

**Figure 5:**
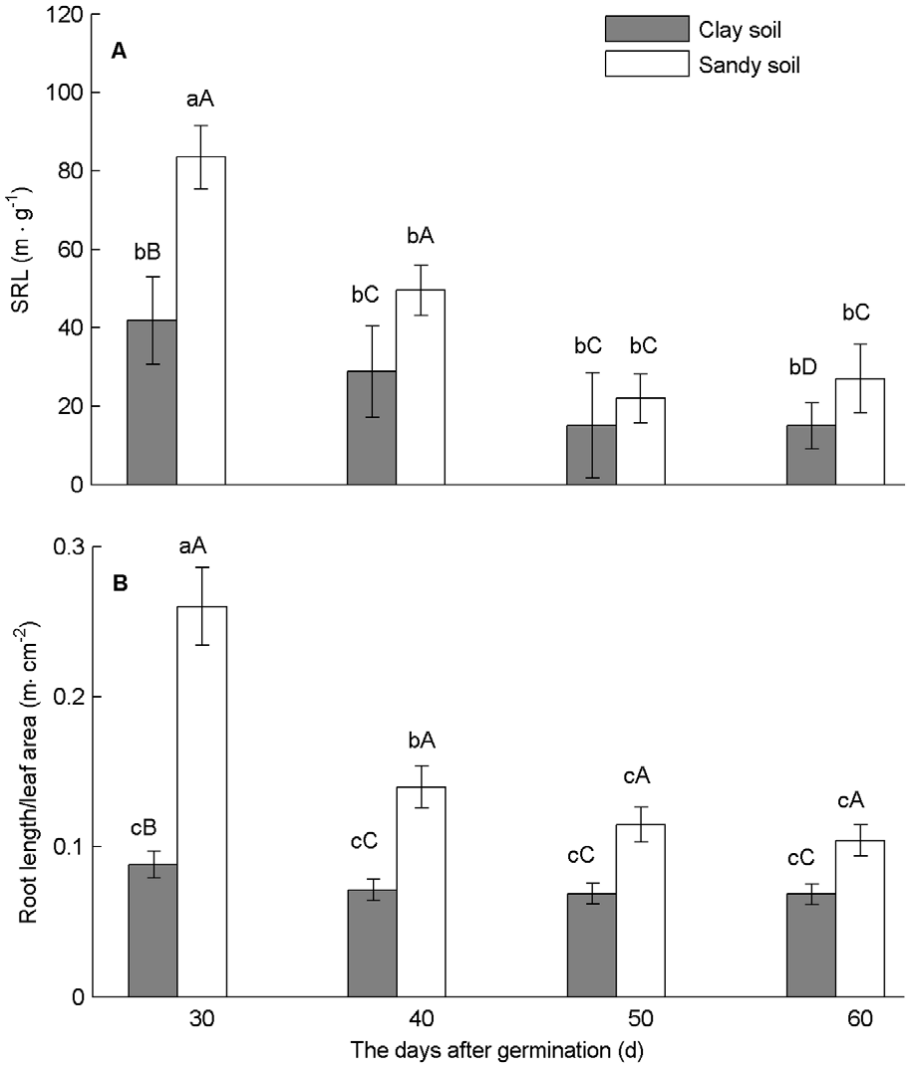
Specific root length and root/leaf ratio in the two soil textures at four growth stages. (A) *SRL*; (B) root length/leaf area ratio. Bars represent average values ± SE, n = 15. Different lower-case and capital letters indicate differences (*p*≤0.05) between growth stages and between soil textures, respectively.

On average, plants in sandy soil allocated 33% less biomass to leaves, and 43% more to roots than in clay soil. However, there was no significant difference in biomass allocation to stems between soil treatments (Fig. 4). The *SRL* and root length/leaf area ratio showed significant differences at each sampling between treatments. The *SRL* and root length/leaf area ratio decreased significantly in sandy soil, but the slight decrease in clay soil was not significant (Fig. 5).

**Figure 6:**
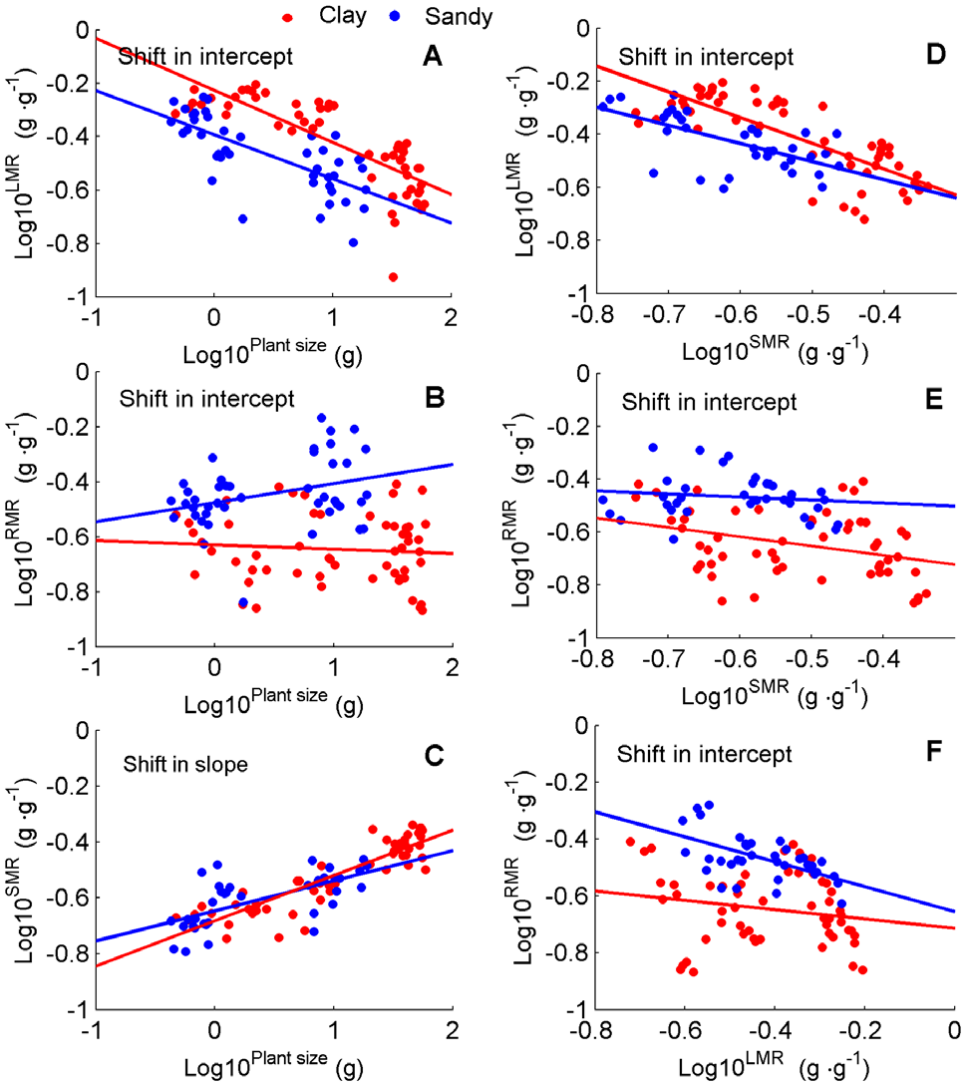
Allometric plots for plant size, *LMR*, *RMR* and *SMR*. Data for individual slopes and intercepts are given in Table 1. The SMA regression (using SMATR package of R) was used to test the slope and intercept heterogeneity at *α* = 0.05 (where slopes or intercepts non-heterogeneous, *P*>0.05) between the two soil textures: (A) Slopes non-heterogeneous, *P* = 0.73; Intercepts heterogeneous: *LMR* higher at a given plant size in clay soil treatment (*P*<0.001). (B) Slopes non-heterogeneous, *P* = 0.47; Intercepts heterogeneous: *RMR* lower at a given plant size in clay soil treatment (*P*<0.001). (C) Slopes non-heterogeneous, *P* = 0.54; Intercepts non-heterogeneous: *SMR* was equal at a given plant size between clay and sandy soil treatment (*P* = 0.13); (D) *LMR* versus *SMR*. Slopes non-heterogeneous, *P* = 0.41; Intercepts heterogeneous, *P*<0.001; (E) *RMR* versus *SMR*. Slopes non-heterogeneous, *P* = 0.33; Intercepts heterogeneous, *P*<0.001; and (F) *LMR* versus *RMR*. Slopes non-heterogeneous, *P* = 0.69; Intercepts non-heterogeneous, *P*<0.001.

The traits changed as plants grew or soil texture affected developmental trajectories or both. Biomass allocation varied with ontogeny, indicating ontogenetic drift. With increased total biomass, stems received an increased proportion of biomass compared to leaves and roots (Fig. 6). When *LMR* decreased significantly with increased plant biomass (slope *b* <0), the plants in clay soil had a higher *LMR* than in sandy soil at a given plant size (intercepts were heterogeneous: *P*<0.001, although, the slopes were non-heterogeneous: *P* = 0.73. Fig. 6A, Table 1). *RMR* also decreased significantly with biomass (*P*<0.001) in clay soil, but increased slightly (slope *b* = 0.069, R^2^ = 0.1) in sandy soil (slopes non-heterogeneous, *P* = 0.47; intercepts heterogeneous, *P*<0.001: *RMR* lower at a given plant size in clay soil treatment than in sandy soil. Fig. 6B, Table 1). In contrast, *SMR* increased significantly (slope *b* >0) with increased biomass (*P*<0.001), with no significant difference between treatments of soil texture (slopes non-heterogeneous, *P* = 0.54; intercepts non-heterogeneous, *P* = 0.13: *SMR* was equal at a given plant size between the two soil treatment. Fig. 6C, Table 1). This phenomenon indicated that soil texture significantly affected biomass allocation to leaves and roots, but did not affect the biomass allocation to stems. In addition, *LMR* is higher at a given *SMR* in clay soil treatment than in sandy soil (Fig. 6D). In contrast, *RMR* is lower at a given *SMR* in clay soil treatment than in sandy soil (Fig. 6E). *RMR* were not significantly different for both soil textures at a given *LMR* (Fig. 6F, see details in Table 1).

**Table 1:** Test for common slope and intercept between the clay and sandy soil textures.

		Slopes (95% CIs), R^2^		H0: slopes are equal	Intercepts (95% CIs)		H0: intercepts are equal	
*Y*	*X*	Clay soil	Sandy soil	LR, *P*	Clay soil	Sandy soil	W, *P*	Type
*LMR*	*M*	−0.24 (−0.29, −0.21), 0.63	−0.23 (−0.29, −0.18), 0.51	0.11, 0.73	−0.18(−0.23, − 1.3)	−0.36 (−0.40, −3.2)	59.62, *P*<0.001	Shift 1
*RMR*	*M*	−0.18 (−0.24, −0.14), 0.1	0.21 (0.16, 0.28), 0.11	0.53, 0.47	−0.45(−0.52, − 0.38)	−0.54 (−0.60, − 0.49)	77.22, *P*<0.001	Shift 1
*SMR*	*M*	0.18 (0.16,0.20), 0.8	0.16 (0.12, 0.21), 0.42	0.36, 0.54	−0.70(−0.73, −0.67)	−0.67(−0.70, −0.64)	2.24, 0.13	Shift 2
*LMR*	*SMR*	−1.23 (−1.46, −1.07), 0.62	−1.08 (−1.41, −0.83), 0.4	0.68, 0.41	−1.05(−1.17, −0.94)	−1.08(−1.26, −0.91)	29.7, *P*<0.001	Shift 1
*RMR*	*SMR*	−1.03 (−1.34, −0.80), 0.14	−0.84 (−1.18, 0.60), 0.1	0.94, 0.33	−1.18(−1.33, −1.04)	−0.97(−1.15, −0.80)	11.56, *P*<0.001	Shift 1
*RMR*	*LMR*	−1.19 (−1.56, −0.91), 0.1	−1.28 (−1.71, −0.97), 0.32	0.16, 0.69	−1.18(−1.4, −0.97)	−1.03(−1.21, −0.85)	29.96, *P*<0.001	Shift 1
Leaf mass	*M*	0.82 (0.78, 0.86), 0.97	0.85 (0.80, 0.90), 0.96	0.98, 0.32	−0.24(−0.29, −0.19)	−0.40 (−0.44, 0.36)	40.51, *P*<0.001	Shift 1
Leaf area	*M*	0.79 (0.76, 0.82), 0.98	0.75 (0.72, 0.78), 0.99	3.71, 0.055	2.02 (1.98, 2.06)	1.93 (1.90, 1.96)	34.15, *P*<0.001	Shift 1
Root mass	*M*	1.00 (0.95, 1.05), 0.97	1.09 (1.03, 1.15), 0.96	4.42, 0.056	−0.65(−0.71, 0.59)	−0.49(−0.53, −0.44)	64.56, *P*<0.001	Shift 1
Root length	*M*	0.71 (0.67, 0.75), 0.97	0.64(0.59, 0.69), 0.96	5.01, 0.054	0.99 (0.95, 1.03)	1.54 (1.52, 1.57)	387.1, *P*<0.001	Shift 1
Stem mass	*M*	1.16 (1.14, 1.18), 0.99	1.11 (1.07, 1.16), 0.99	3.925, 0.05	−0.69(−0.71, 0.67)	−0.65(−0.68, 0.62)	0.47, 0.49	Shift 2
*D*	*H*	0.64 (0.60, 0.68),0.94	0.60 (0.54,0.66), 0.91	5.07, 0.08	−0.23(−0.30, 0.16)	−0.18(−0.26, −0.11)	5.02, 0.08	Shift 2
*SLA*	*M*	−0.043 (−0.013, −0.073), 0.03	−0.0069(−0.0009, −0.012), 0.07	0.45, 0. 12	2.47 (2.38, 2.56)	2.25 (2.21, 2.29)	26.98, *P*<0.001	Shift 1
*SRL*	*M*	−0.44 (−0.53, −0.36), 0.57	−0.50 (−0.59, −0.41), 0.69	1.18, 0.28	1.76 (1.66, 1.86)	1.80 (1.73, 1.87)	0.084, 0.77	Shift 2

**Figure 7:**
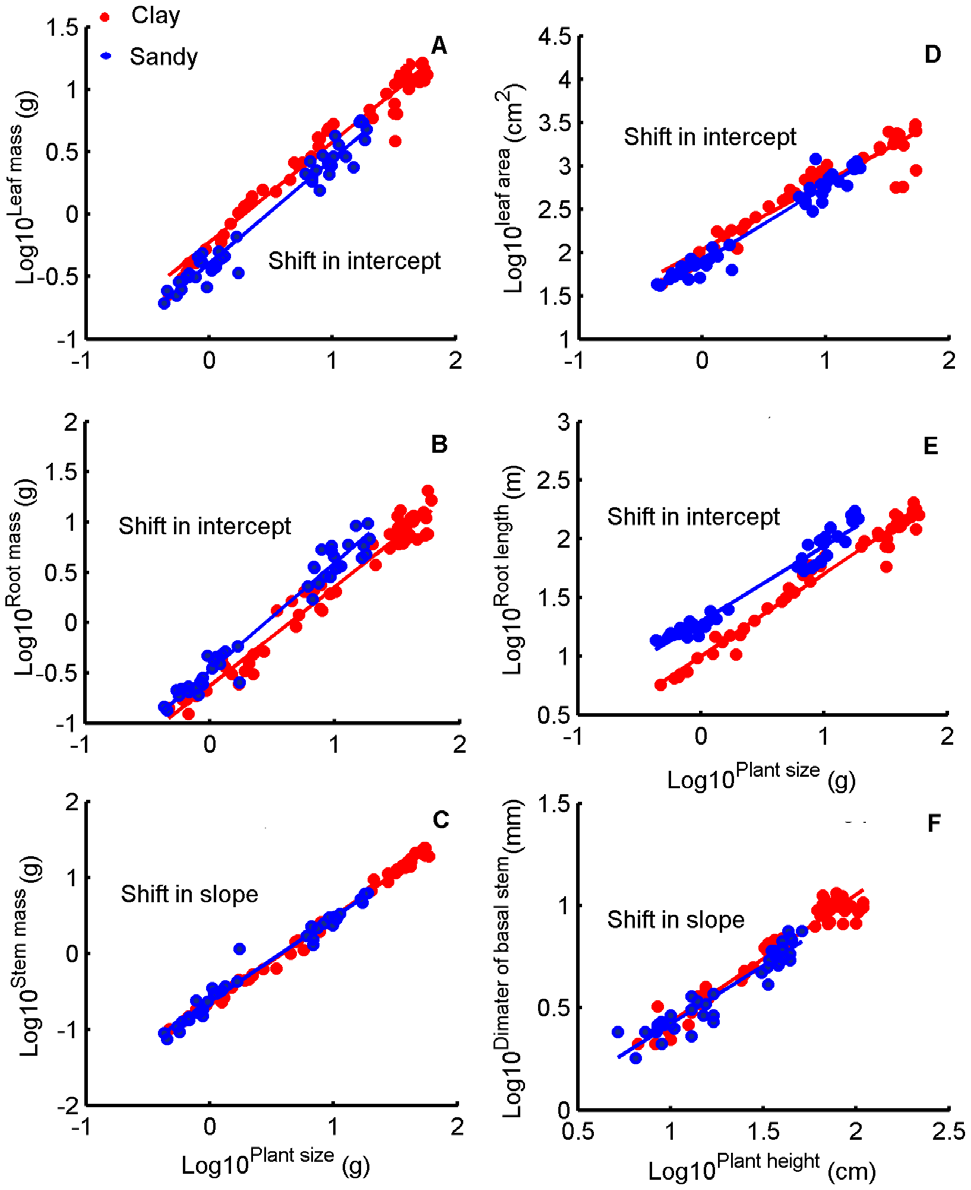
Allometric plots for plant traits. Data for individual slopes and intercepts are given in Table 1. The SMA regression (using SMATR package of R) was used to test the slope and intercept heterogeneity at *α* = 0.05 (where slopes or intercepts non-heterogeneous, *P*>0.05) between the two soil textures: (A) Leaf mass versus plant size. Slopes non-heterogeneous, *P* = 0.32; Intercepts heterogeneous: leaf mass was higher at a given plant size in clay soil treatment (*P*<0.001); (B) Root mass versus plant size. Slopes non-heterogeneous, *P* = 0.056; Intercepts heterogeneous: root mass was lower at a given plant size in clay soil treatment (*P*<0.001); (C) Stem mass versus plant size. Slopes non-heterogeneous, *P* = 0.05; Intercepts non-heterogeneous: stem mass was equal at a given plant size between clay and sandy soil treatment (*P* = 0.49); (D) Leaf area versus plant size. Slopes non-heterogeneous, *P* = 0.055; Intercepts heterogeneous: leaf area was higher at a given plant size in clay soil treatment (*P*<0.001); (E) Root length versus plant size. Slopes non-heterogeneous, *P* = 0.054; Intercepts heterogeneous: root length was lower at a given plant size in clay soil treatment (*P*<0.001); and (F) Diameter of basal stem versus plant height. Slopes non-heterogeneous, *P* = 0.08; Intercepts non-heterogeneous: diameter of basal stem was equal at a given plant height between clay and sandy soil treatment (*P* = 0.08).

**Figure 8:**
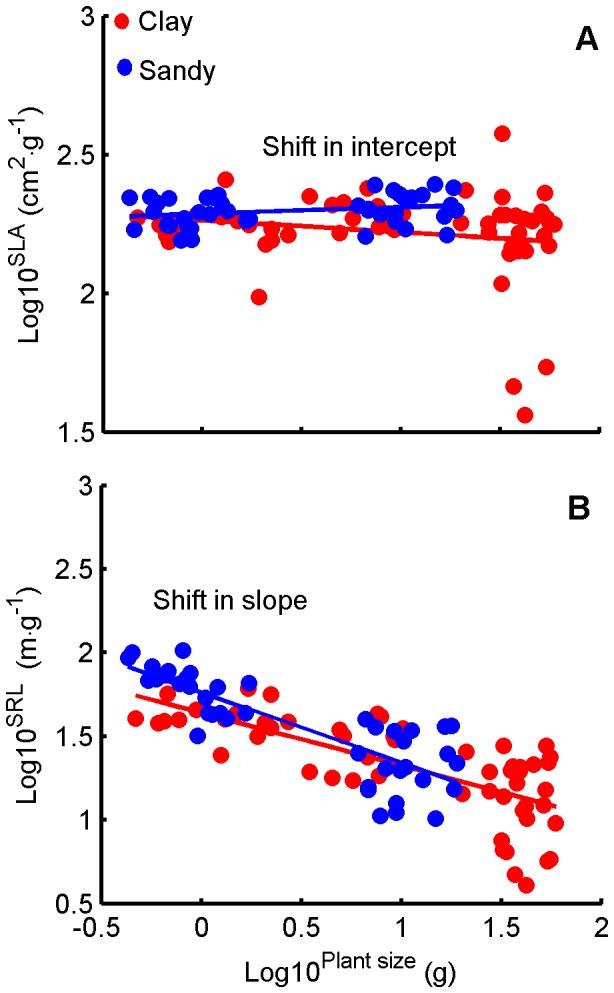
Relationships between plant size and *SLA*, *SRL* in the two soil textures. (A) *SLA* vs. plant size. (B) *SRL* vs. plant size. Data for individual slopes and intercepts are given in Table 1. The SMA regression (using SMATR package of R) was used to test the slope and intercept heterogeneity at *α* = 0.05 (where slopes or intercepts non-heterogeneous, *P*>0.05) between the two soil textures: (A) Slopes non-heterogeneous, *P* = 0.12; Intercepts heterogeneous: *SLA* lower at a given plant size in clay soil treatment (*P*<0.001). (B) Slopes non-heterogeneous, *P* = 0.28; Intercepts non-heterogeneous: *SRL* was equal at a given plant size between clay and sandy soil treatment (*P* = 0.77).

Results for Standardized major axis (SMA) slopes and intercepts fitted within soil textures, corresponding to Figures 6, 7, and 8. Testing for common slopes (where slopes are equal, *P*>0.05) and intercept differences (where no differences in intercept, *P*>0.05). Type is defined in Figure 3. Plant size: *M;* Diameter of basal stem: *D*; Plant height: *H*; Likelihood ratio statistic: LR; Wald statistic: W.

Soil texture significantly changed the developmental trajectories of leaf mass, leaf area, root mass and root length, but did not change the developmental trajectories of stem mass and scaling relationship of basal stem diameter to plant height. This indicated that soil texture only influenced developmental trajectories of some organs (Fig. 7). Some plant leaf traits, such as the relationships of leaf mass with plant size (intercepts heterogeneous, *P*<0.001, although, slopes non-heterogeneous, *P* = 0.32: leaf mass was higher at a given plant size in clay soil treatment. Fig. 7A, Table 1) and leaf area with plant size (Slopes non-heterogeneous, *P* = 0.055; Intercepts heterogeneous: leaf area was higher at a given plant size in clay soil treatment (*P*<0.001). Fig. 7D, Table 1), showed significant differences between soil textures. Moreover, the traits of roots (root mass and length to plant size) also showed significant differences between soil textures (Fig. 7B, 7E, respectively, Table 1). However, the traits of stems differed from that of leaves and roots between treatments. Stem mass increased (b >1) as plants grew, and showed no significant difference in both treatments (slopes non-heterogeneous, *P* = 0.05; intercepts non-heterogeneous, *P* = 0.49: stem mass was equal at a given plant size between clay and sandy soil treatment. Fig. 7C, Table 1). In addition, the scaling relationship of diameter of basal stem to plant height did not significantly differ in both treatments (slopes non-heterogeneous, *P* = 0.08; intercepts non-heterogeneous, *P* = 0.08: diameter of basal stem was equal at a given plant height between clay and sandy soil treatment. Fig. 7F, Table 1). This phenomenon indicated that soil textures did not affect the developmental trajectories of stem mass and the scaling relationships of diameter of basal stem to plant height.

With growth of plants, *SLA* decreased in both treatments (Fig. 8A), and SLA-values remained similar with growth of plants and across treatments. Compared to clay, plants in sandy soil allocated proportionally less biomass to leaves and more to roots, and so led to a substantially greater ratio of roots to leaves, and produced smaller and fewer leaves (Fig. 4). Meanwhile, *SRL* decreased with plant growth in both soils, but decreased more rapidly in sand than in clay soil (Fig. 8B).

**Figure 9:**
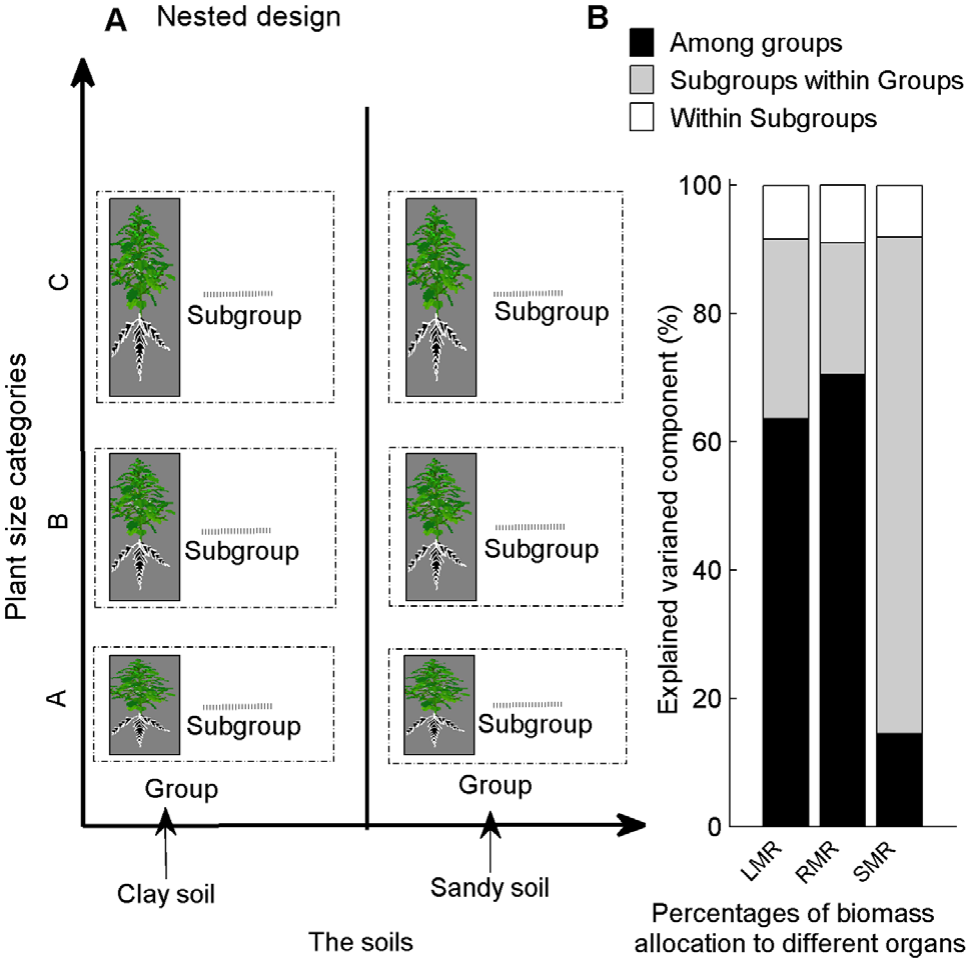
Nested design and variance components of *LMR*, *RMR* and *SMR* based on nested ANOVAs. (A) Nested design. (B) Variance components. Three-level nested ANOVAs: one level was groups, different soil textures; the next level was subgroups, the different plant size categories; and within subgroups, the replications in each plant size category.

The nested ANOVAs of two consecutive plant size categories in both soil textures (see data analysis) indicated that the soil gradients explained on average 63.64–70.49% of the variation in leaf and root mass allocation pattern, and 14.43% of the stem mass allocation pattern. Ontogenetic drift explained 77.47, 20.51 and 28.00% of the variation in the stems, leaves and roots biomass allocation patterns, respectively. For each treatment at each plant size category, the individual differences explained <10% of the variation in total biomass allocation pattern (Fig. 9).

## Discussion

Our data showed the developmental trajectories of leaf and root traits of cotton plants were significantly affected by soil texture (Figs. 6A and 6B, 7 and 8). This indicated that this pattern of biomass allocation differed from that of ontogenetic drift. Meanwhile, the developmental trajectories of stem traits of cotton plants were still governed by ontogenetic drift (Figs. 6C, 7C, 7F). This result suggested that the coordinated plastic response (and thus balanced) of leaf and root traits were the key to understanding how plants sense and respond to environmental gradients (Fig. 6A and 6B, 7 and 8) [23], [24], [48]. In contrast, the developmental trajectories of stem traits seemed unaffected by soil texture, indicating that they were governed by ontogenetic drift (Fig. 6C and 7C and 7F) [24].

The nested ANOVAs of two consecutive plant-size categories in both soil textures indicated that soil texture explained 63.64–70.49% of the variation in root and leaf mass allocation, and that ontogenetic drift explained 77% of the variation in stem mass allocation (Fig. 9). Many studies have concluded that ontogenetic drift caused biomass allocation patterns [2], [9], but they have overlooked the response of biomass allocation pattern to environmental factors, and thus missed mechanisms other than ontogenetic drift.

It is easily understandable that in resource-poor soils, plants develop higher root/shoot ratio than those in resource-rich soils [6], [14]. However, in some cases, delayed plant development may also result in this phenomenon [13], [14]. Some even concluded that this is exclusively a consequence of ontogenetic drift [9], [16]. Our results differed from those conclusions. As pointed out by Moriuchi and Winn [14]: “the biomass allocation of plants in a resource-poor treatment could not be simply due to delayed development”. The nested ANOVAs of the current study distinguished different causes governing the biomass allocations to leaves, roots and stems.

For both soil textures (Fig. 1), all plants were grown under the same evaporative demand, and were well irrigated and fertilized (Fig. 2). Plants were kept free of any physiological stress during growth. Thus soil texture and ontogenetic drift were the only known causes for changes in biomass allocation.

### Response to Soil Texture

In the present study, plants’ absorbing roots in sandy soil were partially exposed to large air-filled soil pores, which created a partial physical discontinuity at the soil–root interface for water movement from soil to roots [34]. This partial discontinuity made the root surface only partially effective in water uptake, and so there would be decreased water uptake per unit root length [34]. This would have probably also reduced nutrient uptake, as the delivery of nutrients by water flow would also be reduced in sandy soil [23], [49]. Consequently, according to the functional equilibrium hypothesis, the allocation to leaves should decrease and that to roots increase [23], [24].

Data of the present study also showed that plants grown in sandy soil allocated more biomass to roots and less to leaves, as shown in previous studies [8], [23] (Figs. 4, 6A and 6B, and 7); and developed higher root/leaf ratios [50] (Fig. 5B), greater root length and less leaf area [34] (Fig. 7) than in clay soil – thus balancing water absorption and consumption. In addition, developmental trajectories of root and leaf traits were also significantly different for the different soil textures. The results of nested ANOVAs (Fig. 9) indicated that development of high activity organs in response to soil texture were better explained by a functional equilibrium between leaves and roots rather than ontogenetic drift.

### Ontogenetic Drift

During experimental periods, sequential samplings showed that most plant traits changed with individual growth. All cotton plants growth along specific trajectories in both treatments; however, different organs showed different characteristics (Fig. 6). *LMR* decreased significantly with increased plant biomass, with plants in clay soil showed a higher *LMR* than in sandy soil. *RMR* also decreased significantly with increased biomass in clay soil, but increased slightly in sandy soil. In contrast, *SMR* increased significantly with increased biomass, and was not affected by soil texture.

We argue that biomass allocation to stems mainly resulted from ontogenetic drift – the data of the current study directly supported this argument. The hydraulic conductance of stem per leaf area did not change for both soil textures (Fig. S1), and the change in biomass allocation to stems contributed little to the water gain of plants in sandy soil [23]. Moreover, stem development is constrained by biomechanics [42]. The data of the current study showed that, in both soil textures, the scaling relationships between basal stem diameter and plant height did not vary (Fig. 7F), and the stem traits against plant size did not change (Figs. 6C and 7C and 7F). This suggested that stem development was constrained by biomechanics rather than soil texture. Therefore, sandy soil led to delays of development only in stems compared to clay soil. This result is consistent with previous findings that (i) a pruning experiment with *Hordeum vulgare* did not cause a shift in biomass allocation to stems [51], (ii) varying soil water availability did not change the *SMR* of plants [23] and (iii) *SMR* did not change with increased nitrogen availability [24].

Ontogenetic drift and response to environment were not mutually exclusive. These two mechanisms co-operate in the development of organs [39]. Therefore, environmental selection could change the developmental trajectories of organs, and could also delay their growth in resource-poor environments. The organ functions and their physiological activities determine which mechanisms govern these processes. Hence, the leaves, roots and stems of cotton plants sensed and responded in different ways. The leaves and roots have higher metabolic activities, higher turnover rates and determining roles in resource acquisition comparing to stems [20], [22], [23], [48], while stems have lower metabolic activities and no roles in resource acquisition in most terrestrial plants [52] – this should result in different responses to environmental gradients [22]–[24].

The current study showed that different organs responded differently to resource gradients. This indicated the need for caution in determining the mechanisms by which plants sense and respond to environmental gradients. For instance, stem trait changes in different environments have frequently been attributed to environmental gradients based on snap-short measurements [14], [53]–[55]. However, the current study indicates that this could also result from delayed stem development in poor environments. Therefore, analyses of development trajectory play an important role in understanding the developing traits of plant organs. It is necessary to distinguish the changes in plant traits due to changes in development trajectory or only developmental delay. This requires comparisons of dynamic processes, rather than snap-short measurements.

In conclusion, we showed that different mechanisms may govern the biomass allocation to different organs. The nested ANOVAs of the present study demonstrated that soil texture mainly governed the biomass allocation to roots and leaves, while ontogenetic drift mainly governed allocation to stems. This finding implies that root/leaf ratio is a good indicator to judge how fast or how well a species will acclimate or adapt to environmental changes. Therefore, it may be a very helpful tool in predicting how successful a species will be in the changed environment of the future [36]. On the other hand, development of supporting organs such as stems can be mainly explained by ontogenetic drift and thus may not be that sensitive to environmental changes. The plant phenotypes and biomass allocation pattern are always driven by genotype–environment interactions [56]. We realize that the current study is based on single species (and single variety) acclimation to soil texture variation and direct conclusion may differ when deal with other species or other environmental factors [11], [57]. However, the basic principle of the current study always stands: the development trajectory is the key here; it tells that whether the change in a plant trait is a response to environment or just developmental delay, or both.

## Supporting Information

Figure S1 **Scaling relationships between stem hydraulic conductance and leaf area.** Statistics are slopes (parameter *b*) and coefficients of determination (*R* ^2^) from SMA regression of leaf area on stem hydraulic conductance within each soil treatment (n ≥22). The SMA regression is also used to test the slope heterogeneity at *α* = 0.05 in soil textures using the SMATR package of R.
(DOC)
