# Supplementary material for: Distinguishing the Biomass Allocation Variance Resulting from Ontogenetic Drift or Acclimation to Soil Texture
Source: PLoS One. 2012 Jul 24;7(7):e41502. doi: 10.1371/journal.pone.0041502 (PMC3404046; doi:10.1371/journal.pone.0041502)
Supplement: Figure S1 — Scaling relationships between stem hydraulic conductance and leaf area. Statistics are slopes (parameter b) and coefficients of determination (R 2) from SMA regression of leaf area on stem hydraulic conductance within each soil treatment (n ≥22). The SMA regression is also used to test the slope heterogeneity at α = 0.05 in soil textures using the SMATR package of R. (DOC) [file pone.0041502.s001.doc]

**Figure S1.**


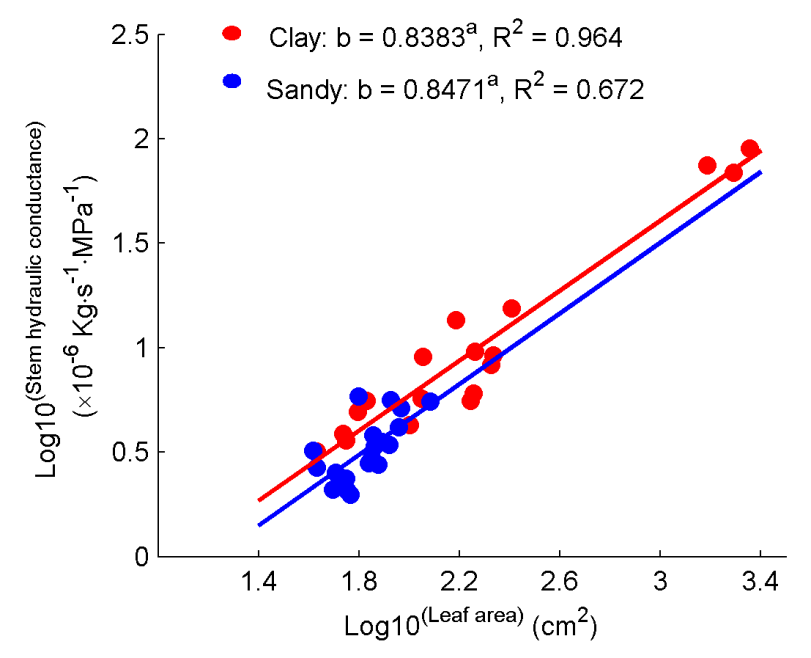


**Figure S1. Scaling relationships between stem hydraulic conductance and leaf area.** Statistics are slopes (parameter *b*) and coefficients of determination (*R*^2^) from SMA regression of leaf area on stem hydraulic conductance within each soil treatment (n ≥ 22). The SMA regression was also used to test the slope heterogeneity at *α* = 0.05 in soil textures using SMATR package of R.

The HPFM (High Pressure Flow Meter, Dynamax Company, USA) was connected to the base of the shoot . Shoots were perfused for 20–40 min at a pressure of 0.5 MPa with deionized water that had been degassed and filtered through a 0.1-mm filter. Shoots were then re-cut 3 cm from the base to remove the plugged section caused by the first perfusion, and perfused again for 10–15 min. Stem conductance was measured by one transient (transient measurement of conductance in HPFM). Transients were performed after removal of leaf blades. At the end of the measurements, the leaves of the shoots were scanned into images, and leaf areas were computed by CI-400 CIAS (Computer Imaging Analysis Software; CID Co., Logan, UT, USA).

The soil textures did not change the scaling relationships between stem hydraulic conductance and leaf area (Fig. S1; F = 0.38, P = 0.54).

**References:**

1. Choat B, Cobb AR, Jansen S (2008) Structure and function of bordered pits: new discoveries and impacts on whole-plant hydraulic function. New Phytol 177: 608-625.

2. Tyree MT, Velez V, Dalling JW (1998) Growth dynamics of root and shoot hydraulic conductance in seedlings of five neotropical tree species: scaling to show possible adaptation to differing light regimes. Oecologia 114: 293-298.

3. Tyree MT, Patino S, Bennink J, Alexander J (1995) Dynamic measurements of root hydraulic conductance using a high-pressure flowmeter in the laboratory and field. J Exp Bot 46: 83-94.

4. Tsuda M, Tyree MT (1997) Whole-plant hydraulic resistance and vulnerability segmentation in *Acer saccharinum*. Tree Physiol 17: 351-357.
